# Relationship between hospital ward design and healthcare-associated infection rates: a systematic review and meta-analysis

**DOI:** 10.1186/s13756-016-0152-1

**Published:** 2016-11-29

**Authors:** Andrea Stiller, Florian Salm, Peter Bischoff, Petra Gastmeier

**Affiliations:** Institute of Hygiene and Environmental Medicine, National Reference Center for the Surveillance of Nosocomial Infections, Charité University Medical Center Berlin, Hindenburgdamm 27, D-12203 Berlin, Germany

**Keywords:** Hospital architecture, Single-patient room, Hand hygiene compliance, Hospital room size, Healthcare-associated infection, Ward design

## Abstract

**Background:**

The influence of the hospital’s infrastructure on healthcare-associated colonization and infection rates has thus far infrequently been examined. In this review we examine whether healthcare facility design is a contributing factor to multifaceted infection control strategies.

**Methods:**

We searched PubMed/MEDLINE, EMBASE and Cochrane Central Register of Controlled Trials (CENTRAL) from 1990 to December 31^st^, 2015, with language restriction to English, Spanish, German and French.

**Results:**

We identified three studies investigating accessibility of the location of the antiseptic hand rub dispenser. Each of them showed a significant improvement of hand hygiene compliance or agent consumption with the implementation of accessible dispensers near the patient bed. Nine eligible studies evaluated the impact of single-patient rooms on the acquisition of healthcare-associated colonization and infections in comparison to multi-bedrooms or an open ward design. Six of these studies showed a significant benefit of single-patient bedrooms in reducing the healthcare-associated colonization and infection rate, whereas three studies found that single-patient rooms are neither a protective nor risk factor. In meta-analyses, the overall risk ratio for acquisition of healthcare-associated colonization and infection was 0.55 (95% CI: 0.41 to 0.74), for healthcare-associated colonization 0.52 (95% CI: 0.32 to 0.85) and for bacteremia 0.64 (95% CI: 0.53 to 0.76), all in favor of patient care in single-patient bedrooms.

**Conclusion:**

Implementation of single-patient rooms and easily accessible hand rub dispensers located near the patient’s bed are beneficial for infection control and are useful parts of a multifaceted strategy for reducing healthcare-associated colonization and infections.

## Background

Preventing healthcare-associated infections, especially with multidrug-resistant bacteria, is paramount for patient safety [1]. In a point prevalence survey conducted between 2011 and 2012 in thirty European countries with 947 acute care hospitals and including 231 459 patients, the European Center for Disease Prevention and Control (ECDC) found a prevalence of 5.7% of healthcare-associated infections (HAI) [2]. There is still insufficient evidence of any correlation between hospital design and infection control. Moreover, the guidelines for healthcare facilities are often vague in their formulation of infrastructural characteristics due to limited evidence in this field of research. While the German Commission for Hospital Hygiene and Infection Control (KRINKO) recommends 10–20% single-patient rooms in a normal care unit, the Facility Guidelines Institute (FGI) recommends performing all patient care in single-patient rooms in its Guidelines for Design and Construction of Hospitals and Outpatient Facilities [3, 4]. According to this, the ratio of single-patient rooms in hospitals is increasing in Europe as well as in North America [5, 6].

Providing hand rub dispensers in patient rooms at the point of care can be a contributing factor for hand hygiene compliance. The proper procedure of hand disinfection has been proven to be one of the most effective infection control measures, however attaining compliance is a challenge [7, 8]. In addition to the number of patients occupying in one single room, the amount of space assigned for each patient within this room is also an important factor. Theoretically speaking, the less space that is provided for patients and healthcare workers within a room, the higher the risk for the transmission of pathogens and for breaches in infection prevention measures possibly leading to an increase in infections. Current directives vary in their recommended square footage for patient rooms: 18.58 m^2^ per bed on critical care units (ICU) in the United States, 25 m^2^ for single-patient rooms or 40 m^2^ for multiple-patient rooms on German ICU’s [4, 9]. The FGI recommends 13.94 m^2^ per patient bed in single-patient rooms and 11.15 m^2^ per patient bed in multiple-patient rooms on critical care units [4]. Germany has not established guidelines for medical/surgical units, whereas the FGI proposes 11.15 m^2^ per patient bed in single-patient rooms and 9.29 m^2^ in multiple-patient rooms [4].

We analyzed available evidence on three crucial aspects of hospital infrastructure: the influence of single-patient rooms, the size of the patient room and the accessibility of antiseptic hand rub dispenser’s location.

## Methods

### Search strategy

The systematic review was done according to the PRISMA guidelines [10] except for registration. We searched for studies that examined the impact of the accessibility of the antiseptic hand rub dispenser’s (AHRD) location inside the patient’s room on hand hygiene compliance and/or healthcare-associated infection rates (topic 1). We also searched for studies, which investigated the influence of single patient rooms (topic 2) and the patient’s room size (topic 3) on healthcare-associated colonization or infection rates, especially caused by multi-drug resistant organisms.

We searched the databases Medline (assessed via Pubmed), EMBASE (assessed via OvidSP) and the Cochrane Central Register of Controlled Trials (CENTRAL). The detailed search strategy used for Medline (Pubmed) for each topic is shown in the Appendix (Tables 2–4).

We screened reviews, systematic review articles and searched the reference lists of eligible articles manually to identify any relevant article not captured by the automated electronic literature search. We searched for full-text articles in English, German, French or Spanish. We included any type of study or trial related to the research questions with a time limit for publication between January 1^st^, 1990 and December 31^st^, 2015. Studies were excluded if they were irrelevant to our research question, noncompliant with the selected language criteria, the full text was unavailable for review despite contacting the authors, if they were duplicate references, publications reporting the same data, reports of outbreaks on individual wards or meeting abstracts. Studies that were conducted in long-term care facilities were also not considered. Finally, letters to the editor, review articles and recommendations were excluded as well.

**Fig. 1:**
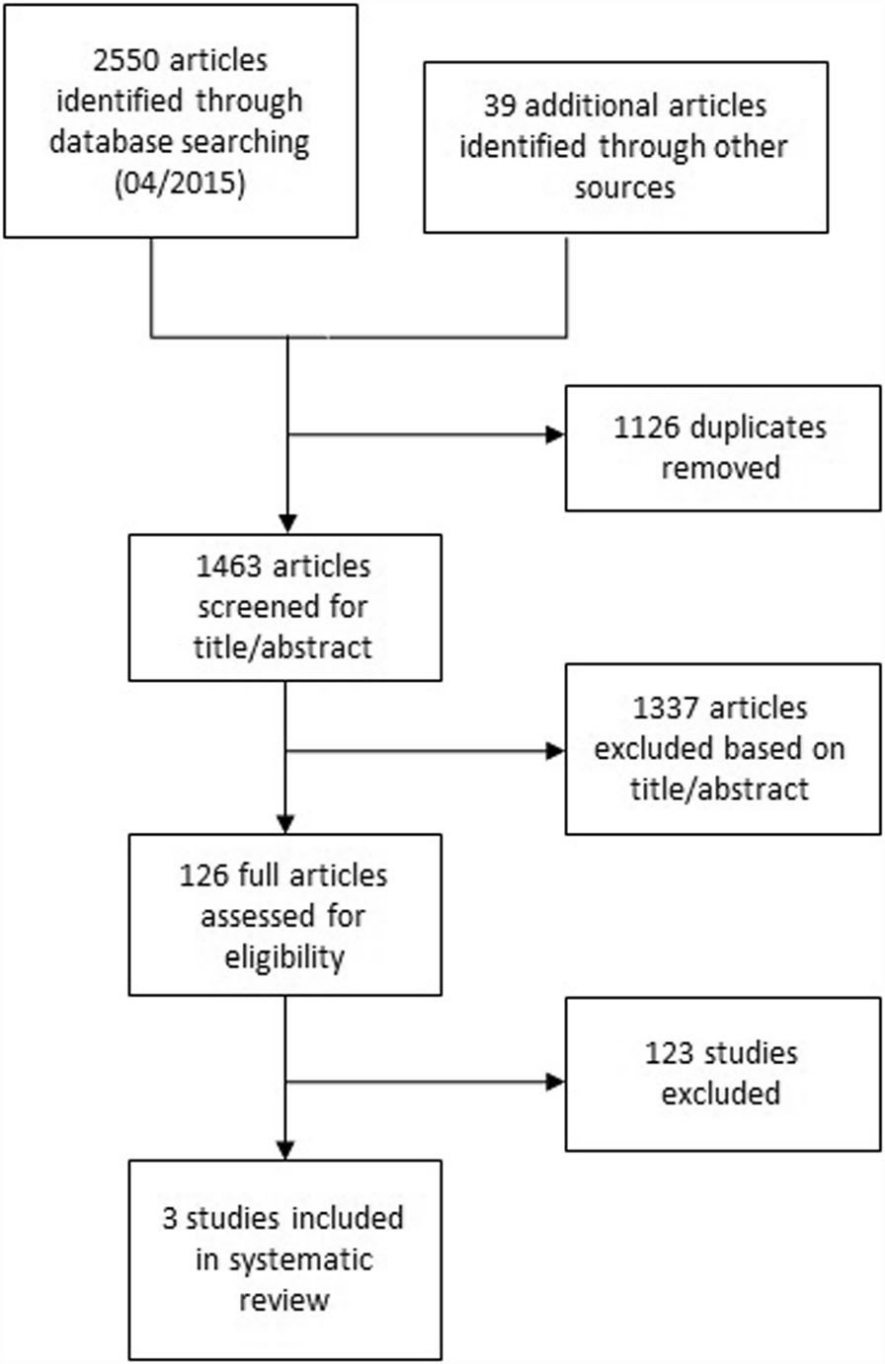
Flow diagram of the study selection process for studies examining the impact of the accessibility of the antiseptic hand rub dispenser’s location on hand hygiene compliance

**Fig. 2:**
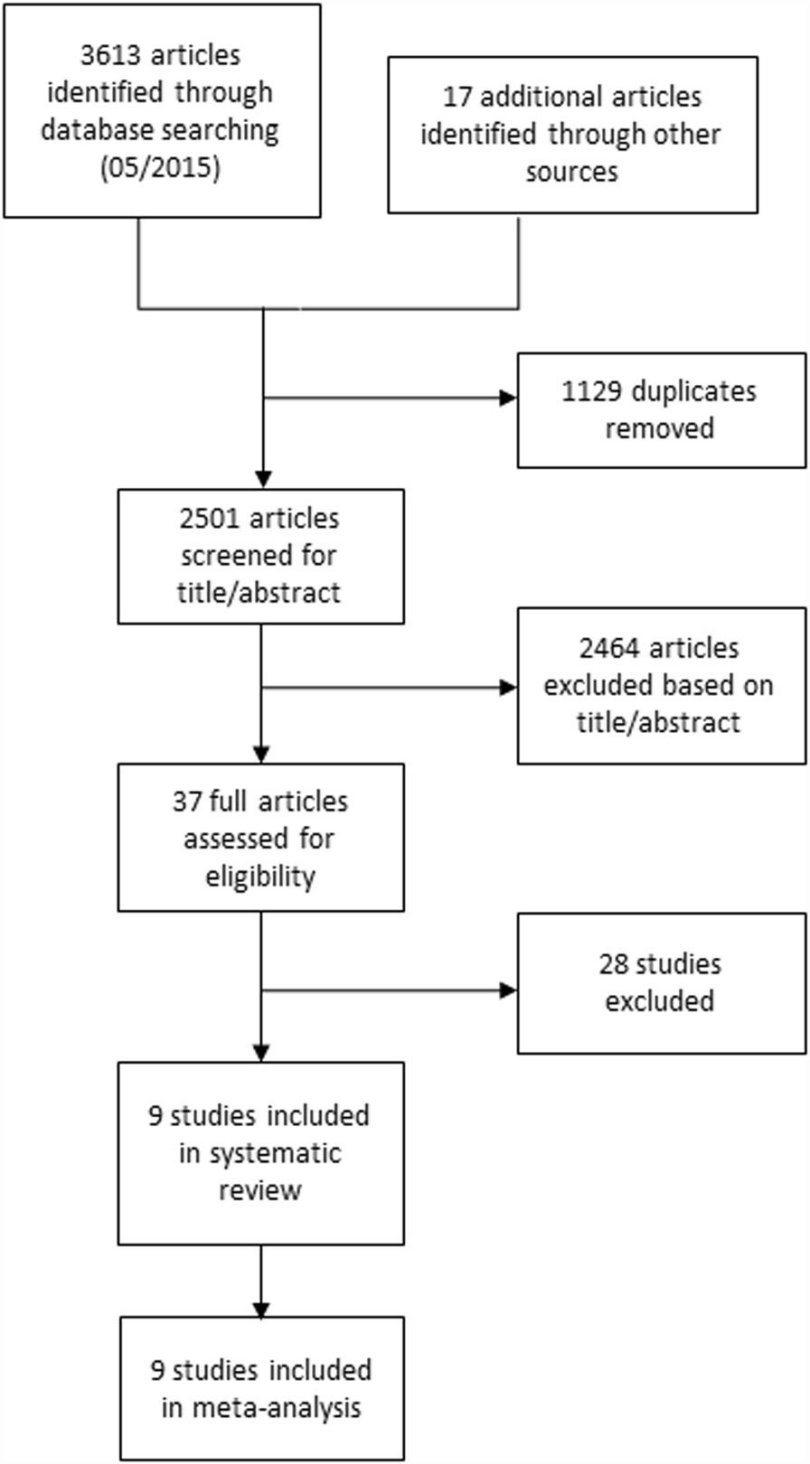
Flow diagram of the study selection process for studies examining the impact of single-patient rooms on healthcare-associated colonization or infection rates

**Fig. 3:**
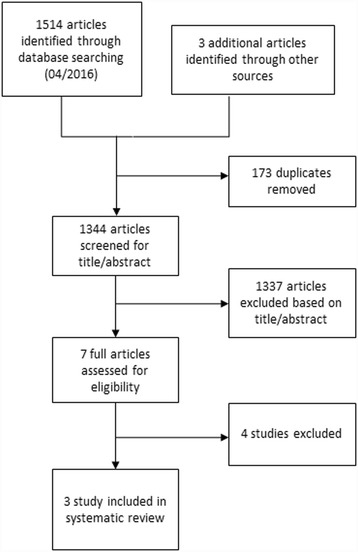
Flow diagram of the study selection process for studies examining the impact of the patient’s room size and physical proximity between patients on healthcare-associated colonization or infection rates

Two authors independently applied the inclusion criteria to the identified articles assessing studies for eligibility. Disagreements between the reviewers were resolved by consensus. We used the ICROMS tool to perform an assessment of the risk of bias of the studies included in the review [11]. The screening and selection process is shown in Figs. 1, 2 and 3.

### Topic 1: Accessibility of the antiseptic hand rub dispenser’s location and hand hygiene compliance

We included studies, in which the accessibility of different locations of the antiseptic hand rub dispenser inside the patient’s room were evaluated with regard to the hand hygiene compliance rate or agent consumption volume. The hand hygiene compliance rate was measured as the percentage of performances counted through direct observation or counted indirectly through agent consumption. Studies investigating hand washing without an antiseptic agent did not meet our inclusion criteria. Additionally, we eliminated studies, which monitored the effect of multimodal intervention programs, or which did not examine the accessibility of the hand rub dispenser inside the patient’s room, for example poster campaigns, staff audits or visual design tools such as signs or lights. Additional studies that we eliminated examined the effect of different dispenser locations associated with an introduction of hand hygiene measures, or investigated dispenser locations outside the patient room, for example on the ward corridor, in the operating theatre, or within the examination room.

### Topic 2: Single-patient rooms and healthcare-associated infections/colonization

We included intervention studies that examine the colonization with multidrug-resistant organisms (MDRO) or infection with any type of pathogen by comparing patient care in single bedrooms with multi-bedrooms or with an open ward design. We excluded surveys of single room isolation, in which single patient rooms or patient cohorting in isolation wards were examined as an infection control measure for already colonized or infected patients. Moreover, we removed studies that discussed bundled interventions, for example additional patient decolonization strategies or healthcare worker education programs. We also excluded a prevalence study, in which the routine use of single patient rooms was analyzed as a variable in a multivariate analysis [12]. We also excluded studies that investigated outcomes other than infection or acquisition of multidrug-resistant organisms, for example psychological effects on patients, economic aspects, and the patient’s length of stay or medication errors.

### Topic 3: Patient room size/proximity between patients and healthcare-associated infections/colonization

While including studies that investigate healthcare-associated colonization with MDRO or infection with any type of pathogen by analyzing the size of a patient’s hospital room and the physical proximity between patients, we excluded studies that examined surface contamination with infectious agents in patient rooms. We also eliminated studies, in which overcrowding was examined as a risk factor, since such studies reported data from outbreak situations or analyzed data irrelevant to our research question.

### Statistical analysis

The identified intervention studies concerning single vs. multi-bedrooms provided sufficient data to allow the calculation of a risk ratio (RR). We used Review Manager (RevMan Version 5.0; The Cochrane Collaboration, 2008) to perform meta-analyses using a random-effects model, if appropriate.

## Results

### Topic 1: Accessibility of the antiseptic hand rub dispenser’s location and hand hygiene compliance

We initially identified 2 550 records. Through manual hand search and by consulting reference lists we identified 39 additional articles. We removed 1 126 duplicates and excluded 1 337 articles that were not relevant to the research question. After application of the inclusion criteria we screened the remaining 126 full articles for eligibility (Fig. 1). 123 studies were excluded because they bundled interventions or discussed the introduction of hand hygiene with an antiseptic disinfectant. Eventually, three studies were included in this review (Table 1) [13–15].

**Table 1:** Characteristics of the selected studies

Study	Setting	Objective	Design	Number of patients		Endpoint
				Intervention:	Control:	
Birnbach et al. [13]	Patient room replica	To investigate the effect of the AHRD’s location on hand hygiene compliance (*n* = 3)	intervention study	Not applicable		hand hygiene compliance
Giannitsioti et al. [14]	Internal medical unit			Not applicable		hand hygiene compliance
Thomas et al. [15]	Surgical ICU			Not applicable		disinfectant consumption
Ben-Abraham et al. [19]	Pediatric ICU	To investigate the association between single bedrooms versus multi bedrooms and healthcare associated colonization or infection rates (*n* = 9)		115	78	Nosocomial infection, bacteremia
Bracco et al. [18]	Surgical ICU			1619	903	Bacteremia Acquisition of MRSA/ Pseudomonas
Ellison et al. [16]	General medical ward			910	604	Infection with or Acquisition of MRSA, CD, VRE
Julian et al. [17]	Neonatal ICU			912	884	CLOS, Acquisition of MRSA
Lazar et al. [21]	Pediatric ICU			1061	3101	Bacteremia
Levin et al. [23]	General ICU			62	62	Bacteremia, Acquisition of any multi-drug resistant organism
McManus et al. [20]	Burn center ICU			914	1605	Bacteremia
Mulin et al. [22]	Surgical ICU			179	135	Infection with *Acinetobacter baumanii*
Vietri et al. [24]	General medical/surgical ICU			130	119	Acquisition of MRSA
Jones et al. [27]	Neonatal ICU/Special Care Nursery	To investigate the association of space per cot and infection rates	Prospective observational study	152	149	Late-onset sepsis
Jou et al. [26]	All hospital wards except ICU	To investigate the association between patient room size and healthcare associated infection rates	Case–control study	75	150	Infection with CD
Yu et al. [28]	All hospital wards except pediatrics	To investigate the risk factors for health-care associated outbreaks of severe acute respiratory syndrome	Case–control study	Not applicable		Severe acute respiratory syndrome

Note: *AHRD* antiseptic hand rub dispenser, *ICU* intensive care unit, *MRSA* methicillin-resistant *Staphylococcus aureus*, *CD Clostridium difficile*, *VRE* vancomycin-resistant enterococci, *CLOS* confirmed late onset sepsis

Birnbach et al*.* utilized a real-size replica of a patient room and observed the hand hygiene compliance of 52 voluntarily participating physicians, who were randomly assigned to one of two groups [13]. The physicians in group 1 examined the patient in a room where the hand rub dispenser was located adjacent to the patient. In group 2, the dispenser was located near the entrance door across the patient’s bed. The compliance rate of the two equally sized groups showed a significant difference (*p* < 0.01): 14 of 26 physicians in group 1 (53.8%) performed hand hygiene with the dispenser positioned adjacent to the patient, while in group 2 only 3 of 26 (11.5%) performed hand hygiene using the dispenser installed at the entrance door.

Giannitsioti et al*.* investigated the appropriate performances of hand hygiene compliance in two internal medicine departments [14]. The patient beds in department A were equipped with an alcohol-based handrub antiseptic on each bed rail while department B provided dispensers on each wall of the wards. For one month, the investigators anonymously recorded opportunities for hand hygiene as well as appropriate uses of antiseptic hand rub. Hand hygiene recording was conducted for a second time period after the bed-rail system had been installed in department B. The study revealed an increased hand hygiene compliance rate in department B following implementation of the bed rail system from 36.4 to 51.5% (*p* < 0.01), while the compliance rate in department A remained almost unchanged (36.4% vs. 35.9%). In a follow-up study conducted six months later, investigators recorded 70 uses of 255 opportunities (27.5%) in department A, in contrast to 80 uses of 302 opportunities (26.5%; *p* < 0.01) in department B over a time period of one month.

Thomas et al*.* investigated the average daily volume use of antiseptic hand rub during three observation periods [15]. They started with a 95-day control period in a 16-bed intensive care unit with eight dispensers, which were located inside the patient rooms as well as outside the patient rooms, i.e., along the floors throughout the ward. During the control period, investigators determined an average daily product use of 188.8 g. Thereafter, a 93-day experimental period was conducted in a newly constructed surgical intensive care unit, in which each bed was equipped with one dispenser. The dispensers were installed on a trapeze-bar apparatus connected directly to the patients’ beds. In this period an average daily use of 294.1 g was measured, which reveals statistical significance in comparison with the control period (*p* < 0.01). In a second experimental period, which continued for 61 days, 36 dispensers were provided in the same locations as during the control period. During this experimental period, an average daily product use of 214.8 g was determined without any statistically significant difference in comparison to the control period (*p* = 0.2).

### Topic 2: Single-patient rooms and healthcare-associated infections/colonization

We identified 3 613 records and located 17 additional articles through hand-searching and by consulting reference lists. After the removal of 1 129 duplicates, we excluded 2 464 articles that were not relevant to the research question. Applying the inclusion criteria, we screened the remaining 37 full articles assessed for eligibility (Fig. 2). We excluded 28 studies on the basis of the criteria explained above. Ultimately, we identified a total of nine studies, in which single-patient bedrooms are compared with multiple patient bedrooms or with an open ward design with regard to the patient’s acquisition of a healthcare-associated colonization with MDRO or infection with any pathogen. These nine studies examined the infectious outcomes of bacteremia, ventilator associated pneumonia, lower respiratory tract infection, gastrointestinal infection, infection of the eye and urinary tract infections (Table 1) [16–24].

The studies were conducted in the United States [17, 20, 24], Canada [16, 18], Israel [19, 21, 23], and France [22]. All but one of the studies were performed in intensive care units. The most frequently used study design was before-intervention and after-intervention observation with or without a control group. The analyzed intervention was the implementation of single patient rooms following ward renovation or moving to a newly built unit. While three studies collected data of the intervention and the control group simultaneously, other studies investigated the same ward before and after the constructional change [16–18]. Additionally, three studies defined hospital-acquired infection and colonization as events occurring ≥ 72 h after admission in contrast to ≥ 48 h after admission [16, 17, 24].

**Fig. 4:**
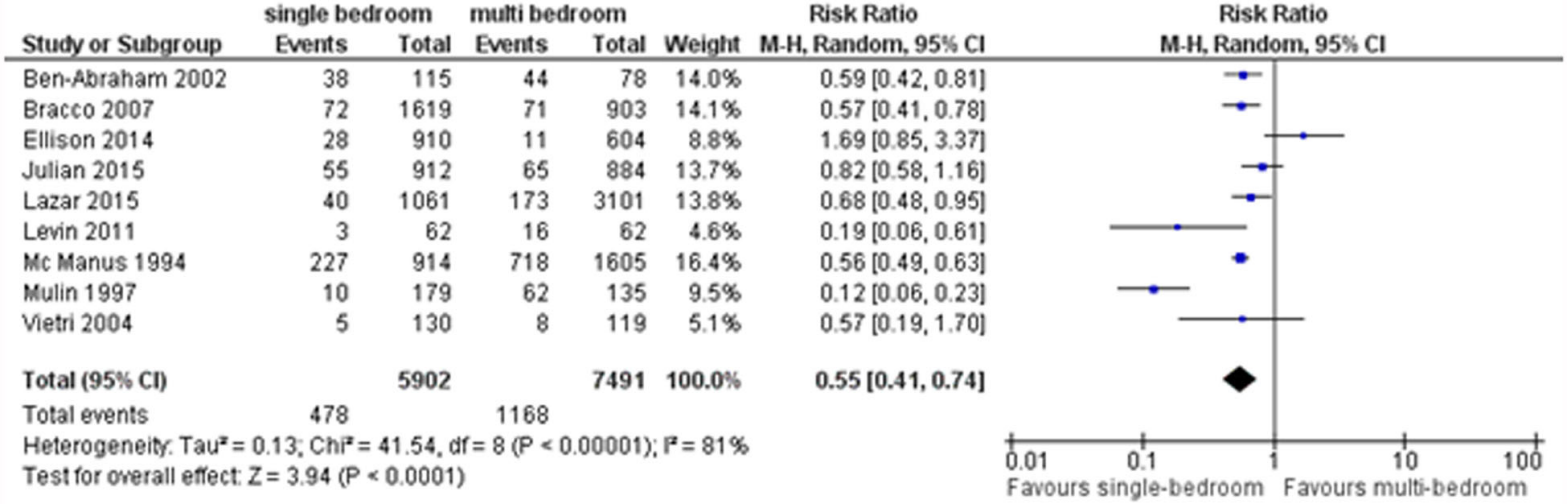
Forest plot of comparison – Studies comparing single- vs. multi-bedrooms, outcome colonization with (multi-)drug resistant pathogens or infection with any pathogen

**Fig. 5:**
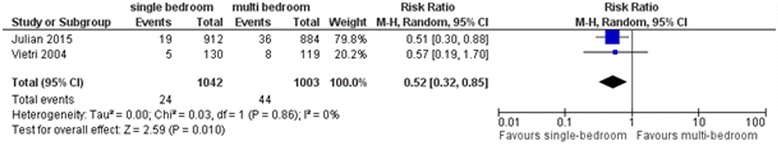
Forest plot of comparison – Studies comparing single- vs. multi-bedrooms, outcome colonization with (multi-)drug resistant pathogens

**Fig. 6:**
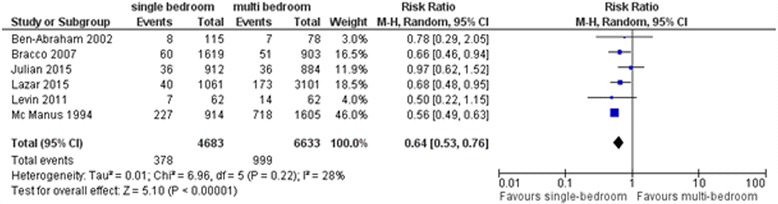
Forest plot of comparison – Studies comparing single- vs. multi-bedrooms, outcome bacteremia

Six studies showed a significant benefit of single-patient bedrooms in reducing the healthcare-associated colonization with MDRO and infection rate [18–23]. However, three studies found that single-patient rooms are neither a protective factor nor a risk factor for colonization and HAI [16, 17, 24]. A meta-analysis of these nine studies showed a significant benefit of single-patient bedrooms in reducing the healthcare-associated colonization and infection rate compared with patient care in multiple patient bedrooms or with an open ward design (RR: 0.55, 95% CI: 0.41 to 0.74, Fig. 4). Separate meta-analysis of two studies which explicitly reported on colonization with MDRO showed a significant benefit of single-patient bedrooms in reducing the healthcare-associated colonization rate (RR: 0.52, 95% CI: 0.32 to 0.85, Fig. 5). Six studies which reported on the outcome of bacteremia were also analyzed separately [17–21, 23]. While three of these six studies revealed a reduced healthcare-associated bacteremia rate associated with patient care in single-patient bedrooms, the other three studies showed no difference in risk. Meta-analysis of these six studies showed a significant benefit of single-patient bedrooms in reducing the healthcare-associated bacteremia rate compared with patient care in multiple patient bedrooms or with an open ward design (RR: 0.64, 95% CI: 0.53 to 0.76, Fig. 6).

Overall, the treatment of patients in a single-patient room seems to have a significant benefit in reducing the healthcare-associated colonization with MDRO and the infection rate with any pathogen compared to treatment in multiple patient bedrooms (Figs. 4, 5 and 6).

### Topic 3: Patient room size/proximity between patients and healthcare-associated infections/colonization

The initial database search resulted in 1 514 records. 173 duplicates were excluded and 1 334 articles were removed since they did not match our inclusion criteria (Fig. 3). We screened the remaining articles and added three data sources located through manual hand search. One study published in 2000 was excluded due to outdated investigation material dating from 1987 [25]. Ultimately, three studies, which met the inclusion criteria, were included in this review (Table 1) [26–28].

While the first study describes the outcome of late-onset sepsis (LOS) on a neonatal intensive care unit in Australia [27], the second study investigates *Clostridium difficile (C. difficile)* infection in an academic medical center in the United States [26]. The third study measures the incidence of severe acute respiratory syndrome (SARS) in 26 different types of hospitals at different locations in China [28].

Jones et al*.* compared rates of LOS before and after the relocation of a neonatal intensive care unit and special care nursery [27]. Data from July to December 2007 was extracted retrospectively for the control group on the old campus and prospectively from July to December 2008 for the intervention group on the new campus. The space per cot increased from 7.6 m^2^ in the old to 17.4 m^2^ in the new intensive care unit and from 4.8 m^2^ in the old to 10.7 m^2^ in the new special care nursery. Investigators determined that 44 of 149 infants (29.5%) had a clinical infection in the control group, in contrast to 34 of 152 infants (22.4%) in the intervention group (Odds Ratio (OR) 0.69, 95%CI: 0.41 to 1.16; *P* < 0.16). Episodes of confirmed clinical infection, as a proportion of all septic episodes, occurred significantly more often in the old campus than in the new campus (OR 0.58, 95%CI: 0.34 to 0.99; *P* < 0.045).

Jou et al*.* evaluated the association between patient room size and acquisition of healthcare-associated *C. difficile* infection [26]. This case–control study surveyed the development of an infection with *C. difficile* during the hospital stay >72 h after admission of patients throughout one year. The control group consisted of patients hospitalized in the same year and was randomly selected. The focus variable was the square footage of the occupied patient room, defined as length x width, at the time of diagnosis. The bivariate analysis showed a significant risk of infection with *C. difficile* associated with a median of 191 square footage [interquartile range (IQR)191-244] compared to 180 square footage (IQR 168–198, OR 2.03, 95%CI: 1.40 to 2.94; *P* < 0.01).

Yu et al*.* conducted a case–control study to analyze the risk factors for health-care associated severe acute respiratory syndrome (SARS) outbreaks among hospital wards in Hong Kong and Guangzhou [28]. Environmental and administrative factors as well as host factors on 48 case wards (SARS patients were admitted and a super spreading event occurred) and 76 control wards (SARS patients were admitted but no super spreading event occurred) were analyzed. The super spreading event was defined as ≥ 3 cases of SARS in a ward during a period of 2–10 days after index patient admission or a cluster of ≥ 3 cases of SARS during a period of 8 days with unknown sources. The univariate analysis demonstrated that the minimum distance between beds of ≤ 1m is a significant risk factor associated with health-care associated outbreaks of SARS (OR 3.71, 95% CI: 1.67 to 8.20; *P* < 0.001). Similarly, the multivariate analysis revealed that a having a minimum distance between beds of ≤1m was a significant risk factor in the hospitals in Guangzhou (OR 5.41, 95% CI: 1.51 to 19.30; *P* = 0.009). However, the association was insignificant at hospitals in Hong Kong (OR 5.13, 95% CI: 0.89 to 29.57; *P* = 0.07). Overall, a minimum distance between beds of ≤1m was a significant risk factor associated with health-care associated outbreaks of SARS at all participating hospital wards (OR 3.36, 95% CI: 1.38 to 8.16; *P* = 0.008).

## Discussion

The purpose of this review was to systematically identify and analyze primary research studies, wherein infrastructural measures were examined as determining factors for infection control. Our research reveals a strong correlation between hospital ward design and healthcare-associated colonization and infection rates. According to our analysis, the implementation of single-patient rooms and the installation of easily accessible antiseptic hand rub dispensers near patient beds are two important facilitators for infection control. Research data about the relationship between the patient room size or the proximity between patients in adjacent beds and the colonization or rates of infection is scarce. We identified three studies, which had entirely different study environments and outcomes. Jones et al*.* investigated the space per cot in a neonatal intensive care unit. They concluded that a significant association exists between a higher square footage per cot and lower late-onset sepsis rates [27]. Jou et al. determined an increased risk of nosocomial *C. difficile* infection in patient rooms with larger square footage [26]. Due to the characteristics of the evaluated pathogen *C. difficile*, it is likely that spores contaminated the surface. This is attributable to the fact that a larger room allows more surface to be contaminated, which leads to an increased transmission risk as cleaning in a larger room could be performed rather inadequately [29]. However, transmission seems to be a minor issue for infection with *C. difficile*. Widmer et al*.* presented a very low rate of transmission in their prospective observational study during an 11-year study period: transmission was detected in 1.3% (6/472) of all contact patients [30]. Another structural aspect was investigated by Yu et al., who investigated the association between the distance between beds and the outcome severe acute respiratory syndrome [28]. They concluded that a minimum of ≥1m between beds is needed to reduce the risk of transmission and thus infection. As this outcome describes a pathogen, which is transmitted via droplet infection, it is questionable to transfer their results to other pathogens. More research is needed on this specific topic to further analyze the implications for infection control measures.

Proper hand disinfection has been proven to be one of the most effective infection control measures. It is quite conceivable that factors improving the compliance rate support the barrier against pathogen transmission [7, 8]. We did not identify any studies investigating on the impact of the location of hand-rub dispensers on healthcare-associated infection rates. However, the results of this review indicate that sustainable improvement of hand hygiene compliance can be supported by locating the hand rub dispenser in the point of care and facilitate its accessibility for healthcare workers [31–33]. Therefore, this review confirms the conclusions made by Kendall et al. who suggest to ensure the availability of the hand rub dispenser in the point of care [33]. Likewise, Zingg et al*.* concluded that a hand-rub dispenser directly in sight of healthcare workers and facilities for hand hygiene at the point of care both improved hand hygiene performance in their systematic review about hospital organization, management and structure for the prevention of HAIs [34] However, as Giannitsioti et al. found out in their follow-up study, a directly accessible dispenser alone may not lead to a sustained compliance improvement [14]. We suggest that easily accessible hand rub dispensers be placed near the patient’s bed at the point of care. This should be combined with other useful compliance improvement measures such as regular staff training and feedback on compliance rates to ensure improved hand hygiene.

The review shows that single-patient rooms are a significant infection control measure in preventing transmission of pathogens from one patient to another due to the fact that no contact transmission can occur either directly from a roommate or indirectly from a healthcare worker taking care of a roommate. Moreover, boundaries that enhance the health care workers’ hand hygiene compliance rate are more firmly established [35]. Conversely, infections can also be caused by the acquisition of pathogens from a prior room occupant [36]. However, a single patient room is considerably easier to clean after the discharge of a patient. Therefore, the risk of environmental contamination could be reduced in comparison to larger and more heavily equipped multi-patient bedrooms.

This review has several limitations. It cannot be ruled out that due to the before-/after-intervention concept the general improvement of medical care over time might have biased the results of some of the studies and consequently biased the results of our meta-analysis (see Figs. 4, 5 and 6). This does not affect three studies [16–18]. Two of these three studies comparing the intervention and control group in the same time period also revealed a benefit in single patient rooms [17, 18]. The study conducted by Ellison et al*.* is found to be the only statistical outlier [16]. Confirmatively, the authors describe what may have compromised the intervention’s potential: shortly after the study began, three single-patient rooms were converted to multi-patient rooms with proximity of 1m between beds. Approximately 50% of the intervention group’s patients stayed in these converted rooms. The considerable heterogeneity in the meta-analysis of studies comparing single vs. multi-bedrooms with the outcome of healthcare-associated colonization/infection could be partially explained by the study design. Hagel et al*.* considered a strong Hawthorne effect on hand hygiene performance, which might attenuate the reported results regarding hand hygiene compliance rate [37].

## Conclusion

The review of the available evidence showed that an easily accessible antiseptic hand rub dispenser enhances the healthcare worker’s hand hygiene compliance rate on acute care units. Furthermore, meta-analyses revealed the benefit of single-patient rooms in reducing the risk of colonization with (multi-)drug resistant pathogens or infections with any pathogen. In order to reduce the risk of transmission in multi-patient bedrooms, the transmission route of the suspected pathogens should be considered. Consequently, a certain distance between patients’ beds should be maintained to prevent droplet transmission and to allow equipment and healthcare workers to move freely between adjacent beds. This leads us to the conclusion that hospital ward design contributes to infection control measures. It is essential to perform further controlled trials to study the effects of an easily accessible AHRD on the hand hygiene compliance rate more precisely and on healthcare-associated infection rates in particular. Well-designed controlled trials investigating the impact of single-patient rooms and the influence of the proximity between patients’ beds on the acquisition of healthcare-associated infections and colonization are imperative.

## Appendix

**Table 2:** Search strategy of MEDLINE via PubMed

#	Searches
Architecture = #1	(“Hospital Design and Construction”[Mesh]) OR room* OR architect* OR ward* OR locat* OR adheren*
Dispenser = #2	(“Hand Disinfection”[Mesh]) OR (“Hand Sanitizers”[Mesh]) OR dispense*
Infection = #3	(“Infection Control”[Mesh]) OR transmiss* OR nosocomial* OR health care associated
Time limit = #4	Limit to “1990/01/01”[PDAT] : “2015/12/31”[PDAT]
Language limit = #5	Limit to (English[lang] OR French[lang] OR German[lang] OR Spanish[lang])
	#1 AND #2 And #3 AND #4 AND #5

Does the location of the hand rub dispenser have an impact on the healthcare worker’s hand hygiene compliance and/or patient’s healthcare-associated infection rate?

**Table 3:** Search strategy of MEDLINE via PubMed

#	Searches
Architecture = #1	(“Health Facility Environment”[Mesh]) OR construct* OR architect* OR design* OR ward*
Single patient room = #2	(“Patients’ Rooms”[Mesh]) OR single OR single patient OR privat* OR single bed
Infection = #3	(“Infection Control”[Mesh]) OR (“Cross Infection”[MeSH Terms]) OR transmiss* OR nosocomial*
Time limit = #4	Limit to “1990/01/01”[PDAT] : “2015/12/31”[PDAT]
Language limit = #5	Limit to (English[lang] OR French[lang] OR German[lang] OR Spanish[lang])
	#1 AND #2 And #3 AND #4

Do single-patient rooms reduce the transmission/infection rate of healthcare-associated infections and/or colonization caused by multi-drug resistant organisms?

**Table 4:** Search strategy of MEDLINE via PubMed

#	Searches
Architecture = #1	(“Hospital Design and Construction”[Mesh]) OR construct* OR room* OR single patient OR architect* OR design* OR ward* OR privat* OR single bed
Size/Proximity = #2	(“Risk Factors”[MeSH Terms]) OR Room size OR square footage OR distance OR proximity
Infection = #3	(“Infection Control”[Mesh]) OR (“Cross Infection”[MeSH Terms])
Time limit = #4	Limit to “1990/01/01”[PDAT] : “2015/12/31”[PDAT]
Language limit = #5	Limit to (English[lang] OR French[lang] OR German[lang] OR Spanish[lang])

Does a higher number of square meters per patient bed decrease the transmission/infection rate of healthcare-associated infections?

## Abbreviations

AHRD: Antiseptic hand rub dispenser
*C. difficile*: *Clostridium difficile*
ECDC: European center for disease prevention and control
FGI: Facility Guidelines Institute
HAI: Healthcare-associated infections
ICU: Critical care units
KRINKO: German commission for hospital hygiene and infection control
LOS: Late-onset sepsis
MDRO: Multidrug-resistant organisms
SARS: Severe acute respiratory syndrome
